# The main phenomena of space crews’ communication as a rationale for the modification of mission control communicative style

**DOI:** 10.3389/fpsyg.2023.1169606

**Published:** 2023-06-12

**Authors:** Natalia Supolkina, Dmitry Shved, Anna Yusupova, Vadim Gushin

**Affiliations:** Russian Federation State Scientific Center, Institute of Biomedical Problems of the Russian Academy of Sciences, Moscow, Russia

**Keywords:** crew communication, content analysis, psychological support, space crews, social support

## Abstract

The article describes the phenomena of communication between space crews and the Mission Control Center studied within the framework of the “Content” space experiment. The experiment was conducted with participation of Russian cosmonauts during ISS-43/44 - ISS-54/55 missions, and a specially developed method of content analysis of crew-to-ground communication was used. It was shown, for instance, that the structure of communication varied significantly depending on the degree of the cosmonauts’ workload and stress-related psychological tension. The main objective of our work presented in this article was discussion of the relationship of the psychological state of cosmonauts, studied on the basis of crew communication content analysis, with their need for social psychological support. The ideas about social psychological support in the context of crew - Mission Control Center (MCC) communication are outlined. Relevant practical recommendations are presented for modifying the communication style of the MCC personnel to psychologically support the crews. The principles and recommendations for effective communication will both provide continuous psychological support to the space crews in orbit and reduce the likelihood of emotional burnout among MCC personnel.

## 1. Introduction

Daily communication between the space crew and the Mission Control Center (MCC) is an integral part of the space flight workflow. Like all professional operations, communications between cosmonauts and MCC have their own schedule and structure. The cosmonaut’s working day begins and ends with a daily planning conference - DPC (morning and evening, respectively), communication during the day is determined by the current daily plan (schedule). The scope of crew-MCC communication is influenced by the substance of the cosmonauts’ tasks, some of which require collaboration with specialists on Earth. The main function of this communication is to inform MCC about the current situation on board and accomplished operations. Thus, the main content of communication in flight is the exchange of information external communicants (ground specialists). The astronauts and cosmonauts should report on the operations they are performing, and, if necessary, clarify the correctness of their understanding of the information received, answer questions from specialists, etc.

As per Soviet occupational psychologist and ergonomist Lomov (1981), any human communication, in addition to the informational component (information exchange), always contains an emotional one (in the space flight, it can be humor, irony, etc.) and an interactive, social role component (e.g., confrontations, commands). That is why the cosmonauts’ verbal messages reflect the features of their emotional response to emerging situations, their mood, motives, as well as group interactions in the crew, etc. Studies of Beregovoy et al. (1993), Kanas et al. (2008), Kanas and Manzey (2008), and Kanas (2015) had shown that cosmonauts and astronauts tend to “drain” the negative emotions accumulated during the flight onto the MCC specialists in order to avoid conflict tension within the crew (transference phenomenon). Thus, the crew-MCC communication is the main source of information about the psychological state of the ISS crewmembers, and its analysis is a standard method of remote psychological monitoring used to identify possible signs of mental distress in cosmonauts and assess its severity (Myasnikov et al., 2001).

Since being in space is associated with constant overcoming of stress caused by adverse factors of a long-term space flight (threat to life and health, sensory and social deprivation, monotony, high responsibility for the operations performed, etc.), the cosmonauts’ speech reflects their stress coping strategies. As Suedfeld et al. (2009, 2015), pioneer of stress coping analysis in astronauts’ speech, stated, the situation of space flight, with its limited or inaccessible instrumental resources, risk to human life and health, and high social responsibility of the actions performed, directly relates to the “excessive” or even “exceeding human resources” level of requirements. Coping strategies are subdivided, according to Lazarus and Folkman (1984) into effective and ineffective ones. According to Suedfeld, effective coping strategies are focused on the inflight problem solving and self-regulation, and ineffective ones are associated with confrontation with partners or avoidance from resolution in order to keep calm.

From the beginning of the era of extended space flights with complicated Mission Protocols, cosmonauts and astronauts have repeatedly expressed the need to optimize communication with the MCC (Bluth, 1981). According to Stuster (2010, 2016) and Noe et al. (2011), the MCC does not always consider existing problems from the astronauts’ point of view, often ignoring it when making decisions. Thus, the “overbearing and over-controlling” style of the MCC in their communication with the crew could cause dissatisfaction among the subjects and an increase in conflict tension in their relationships with specialists on Earth.

Such verbal behavior of the crew can be considered in terms of the well-known “Us and Them” phenomenon manifesting in the group’s confrontation with the outside world, which, according to the group members, does not satisfy their needs completely and cannot be entirely trusted (Battler et al., 2011). The phenomenon of transference of internal negative tension to external communicants, as well as the “Us and Them” phenomenon were later confirmed several times in US space simulations, i.e., in HI-SEAS IV isolation experiment conducted on an analog space research station built on volcanic “Martian and Lunar site” on the island of Hawaiʻi (Goemaere et al., 2019).

Our analysis of the crews’ after flight interviews shows that crew members sometimes try to avoid, intentionally or unintentionally, communication sessions with MCC, considering their informational support as paternalism based on overprotection and tight control. In our opinion, the prevalence of control, judging and advisory functions in the work of MCC operators and specialists allows us to describe their communication style as playing the “Parent” role according to Berne’s transactional model (Berne, 1961; Massey, 1996). This “Parent” is caring about the crew needs, providing astronauts with informational (advice and recommendations), instrumental (additional supplies) and moral (psychological support) resources (according to Lazarus). But sometimes, while too actively demonstrating social position of a “controlling Parent,” and not an equal partner, the MCC specialists unconsciously diminish the role of the crew. This MCC communicative pattern could turn an astronaut on the other side of the communication channel from an effective social position of a capable “Adult” who has adapted at the station and is able to figure everything out on their own - to an overly independent, not quite competent “Child.” It should be especially emphasized that these communicative roles are objectively conditioned by situational context of the crew-MCC interactions. In case of appearance of “Parent–Child” type of communication, according to Berne, disharmony in relations arises, mutual understanding is violated, and the likelihood of conflict increases.

The solution to this problem implies the need both in studies of the relationships between the features of crew-MCC communication and the characteristics of intra- and intergroup interactions, as well as in a significant modification of the MCC communication style. The objective of this article is to make proposals on how to reinforce psychological support in MCC-crew talks, relying on some phenomena detected with the content analysis within the frame of “Content” space experiment.

## 2. Materials and methods

### 2.1. Participants

The subjects were male Russian cosmonauts of ISS 43/44–54/55 flights, who took part in the “Content” space experiment, *N* = 15, age range 40–57. Among these cosmonauts, 7 subjects had an experience of 1 or 2 flights (including the ones incorporated in our studies), and 8 subjects made 3 to 6 flights.

### 2.2. Bioethics and informed consent

The studies involving human participants were reviewed and approved by the Bioethical Commission of the Institute of Biomedical Problems of the Russian Academy of Sciences and fully complied with the principles of the 1964 Declaration of Helsinki.

Each study participant voluntarily signed an informed consent after having the potential risks, benefits and nature of the upcoming study explained to them.

### 2.3. Design of the study

The studies were conducted within the frame of “Content” space experiment involving Russian ISS crewmembers (Gushin et al., 2016). The experiment was dedicated to psycholinguistic analysis of crew-MCC communication.

We studied daily crew-MCC communications during 15 ISS missions with durations from 116 to 340 days (mean 179, median 174).

A corpus of approx. 164,658 statements containing categories of interest were selected from official Roscosmos transcriptions made daily for open (non-confidential) communication channels.

### 2.4. Methods

Quantitative content analysis was used to analyze the cosmonaut’s speech. Content analysis is a systematic, reproducible method of reducing an array of text into a limited number of categories using predefined scientifically grounded coding rules (Krippendorf, 1980; Neuendorf, 2019). The unit of communication analysis is a statement expressing a complete thought (Simonov and Myasnikov, 1982). The system of content analysis categories was developed on the basis of Lazarus and Folkman’s descriptions of effective and ineffective stress coping strategies (Lomov, 1981; Lazarus and Folkman, 1984). Also, additional categories were introduced by Suedfeld et al. (2009, 2015) and Gushin et al. (2016), reflecting the specific features of the cosmonauts’ life and work on orbit (Table 1). According to our initial hypothesis, later confirmed by the obtained results, an increase in the number of statements aimed at social interaction in the crew talks with the MCC, combined with an increase in emotionally charged statements, indicate rising levels of psychological stress.

**Table 1:** Coping-based content analysis categories.

Effective/Adaptive	Ambivalent	Ineffective/Maladaptive
Trust	Seeking social support	Confrontation
Support	Endurance/Obedience	Escape/avoidance
Positive reappraisal		Distancing
Self-control		Claim/Complaint
Initiative		Mistrust
Planful problem-solving (Planning)		Negative emotions
Accepting responsibility		Sarcastic humor
Humor		

The content analysis method was also successfully used to study the crew-MCC communications in a series of IBMP-based model experiments (SFINCSS-99, MARS-500, SIRIUS series) (Gushin, 2003; Supolkina et al., 2021).

### 2.5. Statistical analysis

The data were normalized as rate of statements per week for analysis and processed using SPSS software, methods used were principal component factor analysis (Varimax rotation method with Kaiser normalization), Kruskal–Wallis H test, Wilcoxon W-test, and Mann–Whitney U-test. The nonparametric criteria were chosen due to the fact that in normality check for all data variables (categories of content analysis), a pronounced skewness (to the right) and kurtosis were detected.

## 3. Results

In this chapter, we present a summary of the main results obtained within the frame of “Content” space experiment (Yusupova et al., 2019, 2021, 2022a, 2022b), from 15 ISS missions.

**Figure 1:**
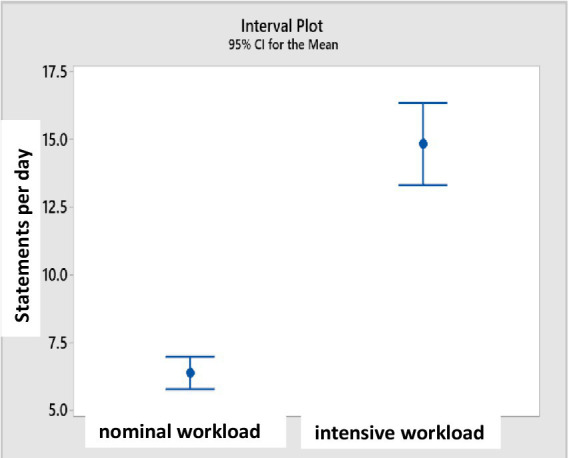
The average number of categorized statements on mission days with standard and intensive workload.

Analysis of the cosmonauts’ conversations with the MCC showed significant differences in the amount of communication depending on the workload. On mission days with a high (intensive) workload, the average number of categorized statements per crewmember was 14.84, on days with a standard (usual) workload was 6.34 (*p* < 0.05) (Figure 1).

In addition to the total amount of communication, the content of the crew-MCC conversations differed significantly on “problem” (high workload) and “calm” (normal workload) mission days. The proportion of categories that characterized effective and ineffective communication differed significantly for 21 categories out of 26. First of all, on problem days, the information exchange increased: cosmonauts were informing ground services about the progress of solving the problems, expressed their need for recommendations. The cosmonauts’ speech was dominated by statements in the categories “planning” (*m* = 1.20), “informing” (*m* = 3.94), “breakdowns/failures” (*m* = 0.29), “problems” (*m* = 1, 37), “search” (*m* = 0.68), as well as “cognitive” (*m* = 1.29) - as a manifestation of the problem solving processes intensification. The number of statements increased in such categories as “effort” (on quiet days *m* = 0.44, on problem days - *m* = 1.29) and “time” (on calm days *m* = 0.62, on problem days *m* = 1.42), which reflects an increase in the psychological stress levels in the crew under high workloads and time constraints. The occurrence of statements in the categories “negative emotions” (almost 6 times: on calm days *m* = 0.12, on problem days *m* = 0.60) and “positive emotions” (on calm days *m* = 0.57, on problem days *m* = 0.94) also significantly increased on problem days. Thus, cosmonauts’ work on problem days is characterized by psychological distress and emotional tension, with a high level of mobilization of psychophysiological resources under the influence of stress factors.

Content analysis of in-flight crew-to-ground conversations showed that the structure of communication (the share of different categories in the total volume of conversations) also varied depending on the degree of workload. With its increase, the number of statements characterizing both adaptive and non-adaptive coping strategies in the cosmonauts’ communication also increases (Table 2).

**Table 2:** Non-adaptive copings and content analysis categories.

Categories	Standard workload	Intensive workload	Value of *p* (Mann–Whitney)
	Mean	Mean	
Negative emotions	0.12	0.60	<0.01
Claim/Complaint	0.35	1.24	<0.01
Confrontation	0.13	0.59	<0.01
Avoiding responsibility	0.15	0.35	<0.01
Self-justification	0.05	0.11	0.013

**Figure 2:**
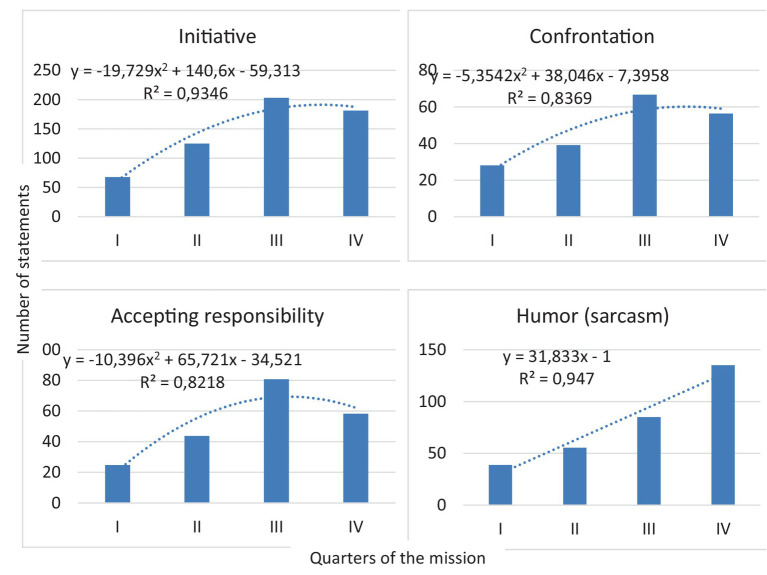
Dynamics of the number of statements by categories of content analysis during space missions.

Among the results obtained, manifestations of the “third quarter phenomenon” discovered for the first time in Russian space studies during the ISS-43/44 – ISS-45/46 expeditions (including the year-long mission) are of particular importance (Figure 2). In some cases, inefficient, from the cosmonauts’ point of view, use of their time, led to the emergence of their counteroffers with negative emotional connotations (category “Confrontation,” the reliability of the polynomial approximation *R*^2^ = 0.837). Thus, a well-trained, experienced crew wanted more independence, which was not always supported by the Earth. That is, during the ISS-43-56 expeditions, “drainage” [“transference” according to Kanas and Manzey (2008) and Kanas (2015)] of negative emotions through communication with the MCC was especially pronounced (Yusupova et al., 2022a,b). These data confirm the results of our American colleagues, principal investigators of the space experiments “Journals” and “Reaction Self Test,” who received similar results in previous studies (Stuster, 2010, 2016; Basner et al., 2014) and consider the “third quarter phenomenon” negatively, as a phenomenon requiring psychotherapeutic correction.

## 4. Discussion

Presented results show the additional stressful impact of the excessive workload during complicated inflight operations (EVA, docking, etc.) and off-nominal situations. Additional stress caused a significant increase in negative emotions and conflict tension in crew communication with MCC which can be interpreted as the transference phenomenon first detected in space flight by Kanas and Manzey (2008) and Kanas (2015).

In the third quarter of the typical long-term space flight (about 6 months), the increase of stress coping manifestations in crew communication was found. We regard this as a possible sign of the “third quarter phenomenon” that was confirmed for polar wintering missions, but not for the long-term space flight yet (Yusupova et al., 2019, 2022a; Kanas et al., 2021). We can explain these changes by accumulation of the negative effects of sensory deprivation, monotony and fatigue during the period when return to Earth seems to be far away. Consecutively, in the fourth quarter it can be mostly gone because of “euphoria” caused by the nearing return back home (Kozerenko et al., 2001; Kozerenko and Holland, 2001) and a lot of new operations while preparing for the landing. This phenomenon was called “the final impulse” by Kozerenko et al. (2001) and Kozerenko and Holland (2001).

Summing up the Results section, we conclude that the stress experienced by cosmonauts is reflected in their talks with MCC, and can be assessed quantitatively with the method described. The level of stress depends on the workload and the period of the flight.

Currently, the inflight psychological provision for the ISS RS crew is carried out in two forms: everyday monitoring of the cosmonauts’ psychological state and implementation of the psychological support measures. Psychological support is a part of the spaceflight medical support operations. The main direction of this type of psychological work is to reduce the negative effect of spaceflight stress factors (monotony, diminishing of social contacts, isolation). The main principle of psychological support is taking into account information about the individual psychological characteristics of the cosmonaut (Burnazyan and Gazenko, 1983).

Since the 1970s and up to our time, the psychological support procedures have remained unchanged: compensation of existing sensory and social deprivation and monotony by providing cosmonauts with emotionally significant information based on the stage of adaptation to space flight, their individual characteristics, and their current socio-psychological status (Kozerenko et al., 2001; Kozerenko and Holland, 2001). Standard psychological support measures include organizing the leisure time of the crewmembers by delivering audio and video content, books, musical instruments, games, etc. via cargo ships, allowing to reconstruct the familiar informational environment. Also, delivery of fresh food not only provides nutrients but allows to enrich the sensory input.

Simulated studies under extended isolation in MARS-500 demonstrated that psychological support in the conditions of social deprivation can be obtained not only by the traditional provision of audio and video contacts with families and friends (Kozerenko and Ponomareva, 2011), but also through regular communication sessions with the MCC specialists (in this study, on-duty medical teams) or communication within the crew (Feichtinger, 2018). That confirms the idea of Cobb (1976) that social support, as a way to cope with stress, is based on “having people you can rely on” (Sarason et al., 1990).

Social support is one of the ways of stress coping (Sheryagina, 2013, 2016). The immediate social environment can help a person to reduce their emotional tension in a problem situation. House and later, other authors proposed to consider social support in terms of four types of resources (House et al., 1988; Kholmogorova et al., 2003, 2014):

- emotional support;
- appraisal support (information relevant to self-esteem);
- informational support;
- instrumental support.

The implementation of certain forms of social support requires the helper to have special communication skills. For example, in the process of communicating with the crew, ground service specialists used not only instrumental and informational support, but also more often provided appraisal and emotional support.

As Cobb (1976) demonstrated, social support in the course of communication leads to an improvement in physical and mental health, and decreases anxiety in recipients. According to the author, social support is actualized when the communicant receives confirmation of their notions through contact, e.g., that they are important, valued, and included in the system of mutual obligations.

That’s why daily crew-MCC contacts not only play the role of instrumental support and information exchange, but can also morally support and motivate the crew, maintain the optimal psychological state and help to cope with stress, especially if they are filled with sincere affiliation (affiliation motivation) (Heckhausen and Heckhausen, 2018). Thus, communication with the MCC not only can, but, in our opinion, should become a part of the psychological support system in long-term space missions. Such a form of psychological support within the frame of professional communication of the cosmonauts saves their time, as well as other instrumental resources of the station (e.g., separate communication channels), since a separate scheduled PS event is not required in this case. Therefore, there is a need in development of a new form of operational psychological assistance to cosmonauts inflight, which can be provided not only by a professional psychologist, but also by any MCC specialist communicating with the crew. Taking into account the peculiarities and conditions of the cosmonauts’ working activities in orbit, we formulated the basic principles for effective communication between MCC specialists and the crew.

First one determines that the objective and content of the talks should be in most cases determined by the crew member, not by the MCC specialist. Since the daily schedule inflight is very tight, and time for each operation is strictly limited, MCC personnel should first give the necessary information to the cosmonaut. The opposite strategy (based on demanding information from the crew, giving directions and orders), according to Stuster (2010, 2016) and Noe et al. (2011), as well as our results, is causing irritation and sometimes open confrontation, especially for cosmonauts with dominating “blaming” communicative style as per Satir’s typology (Satir, 1972; Satir et al., 1991).

Second, to increase the relevance of the MCC-crew communication, it should be conducted in accordance with the data obtained during the parallel process of crew communication monitoring and taking into consideration the cosmonaut’s personality. That means that communication flow and its content should be based on the analysis of the severity of the existing problem situations as well as the actual stress level that the cosmonaut is experiencing. As it’s described above, analysis of the cosmonaut’s coping strategies helps to follow this principle. That means that, according to the data presented in the Results section, this kind of support is especially necessary for the crew during days with excessive workload, including the ones with equipment breakdowns etc., as well as during the third quarter of the flight. That is, cosmonauts and astronauts need moral support while experiencing negative emotions, need understanding when they demonstrate confrontation.

Also, according to Uchino’s (2004) communicative congruence method, it’s necessary to accept the cosmonaut’s communication style as it is, supporting the strengths of the style (for example, knowledge and expertise, initiative, readiness to take responsibility, self-regulation, positive reassessment) in combination with smoothing out its possible weaknesses (such as propensity for conflicts, avoidance of responsibility, distancing). The answers of the specialist must be complementary to the type of communicative reactions of the cosmonauts. That is, it is necessary to “speak their language,” copy their speech features, turns of phrase, etc. In this case, the specialist adjusts to the cosmonaut’s norms, does not try to “correct” him.

Third principle requires from the MCC specialist understanding that, in the cosmonaut-MCC specialist dyad, it is the first one who is in more stressful conditions, and therefore in need for additional psychological (intrapersonal) resources to cope with the problem situations. Following this principle, we need to instill confidence in the cosmonauts; cheer them up, convey positive emotions [emotional support (Birch, 1998)] in order to reduce stress. Therefore, it’s necessary to take the side of the cosmonaut, that is, the ground services and specialists should take responsibility (e.g., for issues being discussed), even if it seems preferable to shift it to the cosmonaut [appraisal support (Birch, 1998)]. If the cosmonaut takes an active position, the leading role should be given to him, and if he is more passive, then the MCC specialist should take the leadership position. Also, accusations, claims and pretensions, criticism should be avoided (appraisal support).

Based on specific signs of emotional tension, an individualized approach to social support *via* communication can be recommended (Table 3). When choosing the style and direction of communication with the cosmonaut, it is proposed to take into account the information from the psychological service about the presence and structure of coping strategies usage, and their focus on resolving or avoiding the problems. According to the data presented in the Results section, cosmonauts with predominantly “blaming” and “placating” styles, who are prone to express stress in their talks, need more support from MCC specialists. Depending on this, MCC operators and specialists may be recommended to use formal (normative-polite behavior), instrumental (providing information support for problem solving), or emotional support (see Table 3). If ineffective coping strategies appear in the cosmonaut’s communication, it is recommended, in order to reduce psychological tension, to actively involve psychologists, schedule additional communication sessions with the cosmonaut’s colleagues, relatives and friends, and, if needed, additional private psychological conferences.

**Table 3:** Manifestations of emotional tension and according types of social support.

Manifestations of emotional tension in cosmonaut’s speech	Types of social support	Examples
No apparent signs of emotional tension, nominal workload.	*Appraisal support*: normative-polite behavior and appraisal support that does not imply emotional involvement and instrumental assistance in solving problems.	“Good morning, glad to hear you” “Thank you for your work”
Appearance of problem-oriented coping strategies and other speech categories reflecting an adaptive reaction to a problem situation.	*Instrumental support:* technical, operational assistance that includes recommendations aimed at solving the problem. The MCC operator gives positive feedback and appraisal support, remains involved in the problem situation, but the feelings of the other communicant are not shared or discussed.	“Let me help you: please try performing this following p. 2 of the manual…” “I contacted the specialist, and she suggested the following…”
Appearance of emotionally-oriented coping strategies and other speech categories reflecting signs of psychological stress, including ineffective strategies (confrontation, complaints, self-justification, avoiding responsibility).	*Emotional support:* recommendations are accompanied by emotional support, demonstration of understanding of the crew’s problems.	“Well, this is a difficult situation, I understand you…” “I would feel the same” “How are you? Are you OK?”
Apparent distress.	*Active support*: assistance in actualization of the cosmonaut’s inner resources. This type of support involves sharing of the crewmembers’ emotions, strengthening their authority and supporting their initiatives. Usually, this form of communication requires special skills.	“Eh, I agree, it’s a difficult situation. The previous crew also had such problems sometimes. I understand that this is unpleasant, but do not worry, we will resolve it together” “I understand you. I support your point, you are right, it’s due to…” “Yes, good idea, let us try this”

## 5. Conclusion

1. The stress experienced by cosmonauts in extended space flight is reflected in their talks with MCC and it can be assessed quantitatively with the content analysis method. The level of stress depends on the workload and the period of the flight.
2. Communication with the MCC can not only provide the crew members with necessary work-related information, but can also become a part of the psychological support system in long-term space missions.
3. Implementation of the principles and recommendations for effective crew-MCC communication could reduce the degree of psychological tension in all participants. That could be achieved by avoiding unnecessary control and critique, giving preferences and responsibilities to the crew members who are experiencing space stress.
4. Based on specific signs of emotional tension, expressed in communication content, an individualized approach to social support *via* MCC talks with the crew can be recommended.

## Data availability statement

The raw data supporting the conclusions of this article will be made available by the authors, without undue reservation.

## Ethics statement

The studies involving human participants were reviewed and approved by Bioethical Commission of the Institute of Biomedical Problems of the Russian Academy of Sciences. The patients/participants provided their written informed consent to participate in this study.

## Author contributions

All authors listed have made a substantial, direct, and intellectual contribution to the work and approved it for publication.

## Funding

The study was supported by the Russian Academy of Sciences, research topic 63.2.

## Conflict of interest

The authors declare that the research was conducted in the absence of any commercial or financial relationships that could be construed as a potential conflict of interest.

## Publisher’s note

All claims expressed in this article are solely those of the authors and do not necessarily represent those of their affiliated organizations, or those of the publisher, the editors and the reviewers. Any product that may be evaluated in this article, or claim that may be made by its manufacturer, is not guaranteed or endorsed by the publisher.
